# Curcumin protects rats against gentamicin-induced nephrotoxicity by amelioration of oxidative stress, endoplasmic reticulum stress and apoptosis

**DOI:** 10.1080/13880209.2022.2037663

**Published:** 2022-02-21

**Authors:** Pongrapee Laorodphun, Rada Cherngwelling, Aussara Panya, Phatchawan Arjinajarn

**Affiliations:** a Ph.D.’s Degree Program in Biology (International Program), Faculty of Science, Chiang Mai University, Chiang Mai, Thailand; b Department of Biology, Faculty of Science, Chiang Mai University, Chiang Mai, Thailand; c Department of Physiology, Faculty of Medicine, Chiang Mai University, Chiang Mai, Thailand; d Research Center in Bioresources for Agriculture, Industry and Medicine, Chiang Mai University, Chiang Mai, Thailand

**Keywords:** Oxidative damage, natural antioxidant, renoprotective

## Abstract

**Context:**

Gentamicin (GM) is an aminoglycoside antibiotic which is commonly used against Gram-negative bacterial infection; however, serious complications including nephrotoxicity could limit its clinical use.

**Objective:**

The present study examined the protective effects of curcumin (CUR) on endoplasmic reticulum (ER) stress-mediated apoptosis through its antioxidative property in GM-induced nephrotoxicity in rats.

**Materials and methods:**

Male Sprague-Dawley rats (*n* = 3) were divided into six groups to receive normal saline (control), GM (100 mg/kg/day), co-treatment with GM and CUR (100, 200 and 300 mg/kg/day) and CUR (200 mg/kg/day) alone for 15 days by gavage feeding. Then, the renal function, kidney injury as well as oxidative stress, antioxidative markers and ER stress-mediated apoptosis were evaluated.

**Results:**

Pre-treatment of CUR rescued the nephrotoxicity in GM-treated rats. Several nephrotoxicity hallmarks were reversed in the CUR-pre-treatment group. At the dose of 200 mg/kg/day, it could significantly lower serum creatinine (from 0.95 to 0.50 mg/dL), blood urea nitrogen (from 35.00 to 23.50 mg/dL) and augmented creatinine clearance (from 0.83 to 1.71 mL/min). The normalized expression of oxidative stress marker, malondialdehyde was decreased (from 13.00 to 5.98) in line with the increase of antioxidant molecules including superoxide dismutase (from 5.59 to 14.24) and glutathione (from 5.22 to 12.53). Furthermore, the renal ER stress and apoptotic protein biomarkers were lowered in CUR treatment.

**Discussion and conclusions:**

Our findings pave the way for the application of CUR as a supplement in the prevention of nephrotoxicity and other kidney diseases in the future.

## Introduction

Nephrotoxicity can be a result of hemodynamic changes due to toxic effect of medications and chemicals. Most drugs found to cause nephrotoxicity exert toxic effects by one or more common pathogenic mechanisms (Al-Naimi et al. 2019). Gentamicin (GM) is an aminoglycoside antibiotic used in the treatment of serious infections which caused by Gram-negative bacteria (Balakumar et al. 2010). Although GM is the most frequently used aminoglycoside because of its broad-spectrum activity, rapid bactericidal action and low cost, serious side effects such as nephrotoxicity have limited the clinical use of the drug (Edson and Terrell 1999; Lopez-Novoa et al. 2011). Several reports indicated that up to 30% of patients who received GM for more than seven days showed signs of nephrotoxicity characterized by increases in the serum level of creatinine and blood urea nitrogen (BUN), with a decrease in renal clearance, changes in body weight and urine volume and severe proximal renal tubular necrosis, which eventually result in renal dysfunction and failure (Soliman et al. 2007; Abdel-Raheem et al. 2009).

In recent years, newer specific and sensitive tubular injury markers such as kidney injury molecule-1 (KIM-1) and neutrophil gelatinase-associated lipocalin (NGAL) have received increasing attention (Alnajjar et al. 2020). KIM-1 is a recently discovered proximal tubule apical transmembrane protein (Tanase et al. 2019) as well as NGAL is a 25 kDa protein of the lipocalin family expressed on tubular cells (Singer et al. 2013). Additionally, the membrane proteins which are expressed in glomerular podocytes including nephrin and podocin become useful to detect glomerular injury (Bonventre 2008; Chen et al. 2015). Higher expression of KIM-1 and NGAL but lower expression of nephrin and podocin was seen in response to various pathologic states or toxicants (Bonventre 2008; Singer et al. 2013).

Although the exact mechanism underlying GM-induced nephrotoxicity is still not well understood, recent studies have demonstrated that reactive oxygen species (ROS) are involved in GM, inducing renal damage, and are important mediators in GM-induced nephrotoxicity (Pedraza-Chaverri et al. 2000; Cuzzocrea et al. 2002). Gentamicin has been shown to enhance the production of superoxide anions, hydroxyl radicals and hydrogen peroxide from renal cortical mitochondria (Said 2011; Tavafi and Ahmadvand 2011). These ROS induce cell injury and death, which reflects both its impaired structure and function (Nitha and Janardhanan 2008; Stojiljkovic et al. 2012). Recent studies have also postulated that an increase in ROS production, and subsequently oxidative stress, generated by GM are associated with endoplasmic reticulum stress (ER stress) (Crow et al. 2004).

ER is a eukaryotic cellular organelle that plays an important role in protein folding, export and processing and the regulation of Ca^2+^ homeostasis and apoptosis (Berridge 2002; Rashid and Sil 2015). It has been shown that accumulation of GM in the ER can lead to ER stress, and activation of the unfolded protein response (UPR) (Zhang et al. 2006). The activation of the UPR triggered the synthesis of ER chaperone GRP78 (78-kDa glucose regulated protein), which is also called BiP, the regulator of the endoplasmic reticulum (ER) stress response (Wang et al. 2009). Additionally, ER stress also leads to increased cytosolic calcium levels, which induce the activation of calpain, and thereafter caspase-12 activation (Nakagawa and Yuan 2000; Fribley et al. 2009). Moreover, sustained or severe ER stress causes an increase in the induction of transcription factor C/EBP homologous protein (CHOP), which in turn leads to Bax/Bak and caspase activation (Szegezdi et al. 2006). Accordingly, therapeutic agents that inhibit and/or reverse these effects have gained extensive attention.

Curcumin (CUR) is a major yellow phenolic pigment of turmeric that is extracted from rhizome of *Curcuma longa* L. (Zingiberaceae), a spice widely cultivated in tropical countries in south and southeast Asia, such as China, India and Thailand (Wanninger et al. 2015). It is commonly used as a spice in curries, food additives, and dietary pigments (Ammon and Wahl 1991). Numerous studies have shown that CUR has excellent antioxidant and anti-inflammatory properties. It has the ability to inhibit free radical generation, scavenge ROS and induce an antioxidant response. Moreover, CUR has also exerted renoprotective effects in several experimental models, including diabetic nephropathy, chronic renal failure, ischaemia and reperfusion; it also combats nephrotoxicity protecting against renal injury from oxidative stress (Trujillo et al. 2013). Interestingly, CUR treatment helps protect against acute myocarditis by inhibition of cardiac oxidative and ER stress-mediated apoptosis (Mito et al. 2011). Our aim here, therefore, was to study the effect of CUR on ER stress-induced apoptosis in GM-induced nephrotoxicity in a rat model.

## Materials and methods

### Chemicals and reagents

Gentamicin was obtained from the Pharmaceutical Organization, Inc. (Bangkok, Thailand). Curcumin was purchased from the Cayman Chemical Company (Ann Arbor, MI). Biochemical kits for TBARS and glutathione (GSH) assay were obtained from the Cayman Chemical Company (Ann Arbor, MI). Superoxide dismutase (SOD) assay kit was purchased from BioAssay Systems (Hayward, CA). All other chemicals and reagents were purchased from a commercial source at the analytical pure grade.

### Animals

Male Sprague-Dawley rats weighing between 180 and 200 g were obtained from the National Experimental Animal Center (Nakhon Pathom, Thailand) and allowed one week of acclimatization upon arrival to the animal facilities. The animals were housed at a constant temperature (22 ± 2 °C), humidity (55%) and 12 h day/night cycle with a standard pellet diet and tap water *ad libitum*. The animal facilities and protocols were approved by the Laboratory Animal Care and Use Committees at the Faculty of Medicine, Chiang Mai University, Chiang Mai, Thailand (permit number: 2/2557).

### Experimental design

**Figure 1.**
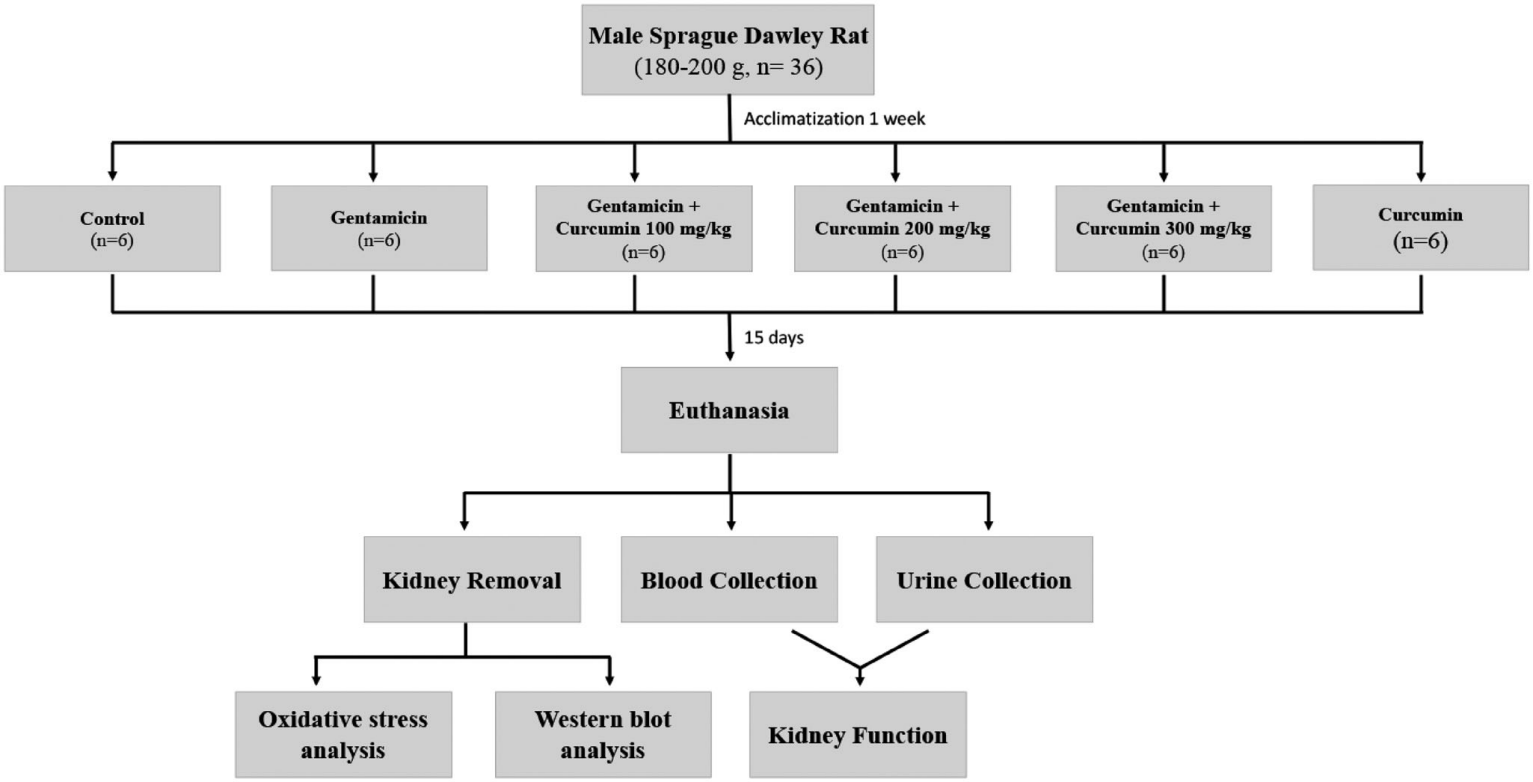
Flowchart depicting the study design.

Thirty-six rats were randomly divided into six groups (*n* = 6 rats/group). In the control (CON) group, the rats were intraperitoneally (i.p.) injected with normal saline. Rats in the GM group were i.p. administered with GM 100 mg/kg/day (Abdelmoaty and Imam 2019). Rats in the GM plus CUR received GM (100 mg/kg) together with CUR at doses of 100, 200 or 300 mg/kg/day (GM + CUR 100, GM + CUR 200, GM + CUR 300, respectively). The dosage of each supplement was chosen according to prior study (Abdelmoaty and Imam 2019). Curcumin was dissolved in deionized water (1 mL) and was administered via oral gavage 30 min before GM injection. Rats in the CUR group received CUR alone at a concentration of 200 mg/kg/day. All treatments were administered for 15 consecutive days. The experimental design of the study is shown in Figure 1.

### Blood and renal tissue sampling

After the last injection of GM, the animals were immediately kept in individual metabolic cages to collect 24 h urine for the assessment of renal function. The animals were killed with an i.p. injection of pentobarbital (100 mg/kg) and blood was subsequently collected from the abdominal aorta. Serum was separated by centrifugation for biochemical estimations. Thereafter, the abdomen was opened through a long midline incision and the kidneys were flushed with an ice-cold HEPES-sucrose buffer (containing 250 mM sucrose, 10 mM HEPES, pH 7.42–7.44) through the abdominal aorta. The kidneys were removed carefully and weighed after decapsulation. Then, the renal cortex was separated and prepared for malondialdehyde (MDA), SOD and GSH level determination and Western blot analysis.

### Renal function assessment

Serum levels of BUN and creatinine, as well as urine creatinine, were measured with an automatic analyser (Reflotron Plus; Roche Diagnostics, Barcelona, Spain). Creatinine clearance (Ccr) was calculated using the standard formula *C*=*U*×*V*/*P*, where *C* is the Ccr, *U* is the urine concentration of creatinine in mg/dL, *V* is the urine flow rate per minute in mL/min and *P* is the plasma concentration of creatinine in mg/dL (Cockcroft and Gault 1976).

### Malondialdehyde assay

To determine the level of renal oxidative stress, the renal cortical MDA level was assayed by colorimetry as previously described (Ohkawa et al. 1979). The samples were homogenized and centrifuged at 1600×*g* at 4 °C for 10 min. The supernatant was assayed with a TBARS colorimetric assay kit (Cayman Chemical Company, Ann Arbor, MI) and the results were expressed as nmol/mg protein.

### Superoxide dismutase activity assay

SOD activity was measured in the renal cortical tissue using a commercial EnzyChrom Superoxide Dismutase assay kit (BioAssay Systems, Hayward, CA). Enzymatic activity was expressed in U/mL.

### Total glutathione assay

Renal cortical GSH was determined by a colorimetric method using a commercial Quantichrom Glutathione assay kit (BioAssay Systems, Hayward, CA) according to the manufacturer’s procedures. The level of GSH was defined as mmol/mg protein.

### Preparation of samples and Western blot analysis

Kidney cortex samples were homogenized in mammalian cell lytic buffer (Sigma-Aldrich, St. Louis, MO) containing a protease inhibitor (Roche, Indianapolis, IN) and centrifuged at 10,000×*g* for 15 min at 4 °C. After centrifugation, the supernatants were removed and the whole cell lysate fraction was labelled. The whole cell lysate fraction was further centrifuged at 100,000×*g* for 2 h at 4 °C to generate a membrane (pellet) fraction. Equal volumes of each fraction were loaded and electrophoresed onto 10%, 12% and 15% SDS-PAGE and transferred to PVDF membrane (Millipore, Billerica, MA). Next, membranes were blocked with 5% non-fat dry milk in PBST or TBST, or other blocking solution for 1 h at room temperature, followed by probing with primary antibodies overnight at 4 °C. The antibodies against NGAL, caspase 12 and Bax were obtained from the Millipore Company (Boston, MA). Antibodies against calpain2, cytochrome C, Bcl-2, cleaved caspase 3, cleaved caspase 7, CHOP, TGF-β, Na^+^/K^+^-ATPase and actin were obtained from Cell Signaling Technology (Boston, MA). Antibodies against GRP78, KIM-1, nephrin and podocin were received from Abcam (Cambridge, UK). Membranes were washed and then incubated for 1 h at room temperature with HRP-conjugated anti-rabbit or anti-mouse secondary antibody (Amersham, Arlington Heights, IL). Thereafter, proteins were detected by ECL enhanced chemiluminescence agent (GE Healthcare, Buckinghamshire, UK) on Hyperfilms (GE Healthcare, Buckinghamshire, UK) and analysed using Image J (NIH Image, Bethesda, MD) analysis software. Protein levels were normalized by the corresponding level of β-actin.

### Statistical analysis

The data were analysed using Statistical Package for the Social Sciences (SPSS) Statistics for Windows, Version 17.0 (2008; SPSS Inc., Chicago, IL). All data were reported as mean ± SEM. For comparison between multiple treatments, one-way ANOVA followed by Fisher’s least significant difference (LSD) test was used. *p* Value <0.05 was considered statistically significant.

## Results

### Effect of curcumin pre-treatment on body weight, kidney weight and kidney weight per body weight ratio and renal function parameters

The mean body weights of all experimental groups of rats were comparable at the beginning of the experiment. After the 15 days of GM injection, it caused a marked reduction of body weight to 273 ± 9 g when compared with CON group (358 ± 2 g) (Table 1). Furthermore, the GM-treated rats had renal hypertrophy judged by the significant increases in kidney weight (from 1.40 ± 0.04 to 2.00 ± 0.06 g) and kidney weight per body weight ratio (from 3.92 ± 0.11 to 7.41 ± 0.44 mg/g). Comparing to the CON group, the rat with GM treatment significant increased the serum BUN from 21.17 ± 0.83 to 35.00 ± 0.73 mg/dL and creatinine levels from 0.46 ± 0.02 to 0.95 ± 0.04 whereas urine creatinine and Ccr were apparently decreased from 60.26 ± 5.23 to 29.01 ± 2.23 mg/dL and from 1.82 ± 0.10 to 0.83 ± 0.09 mL/min, respectively. Interestingly, GM-treated rats receiving CUR at doses of 100, 200 and 300 mg/kg showed significant increases in body weight to 333 ± 6, 346 ± 8 and 343 ± 4 g, respectively. Renal hypertrophy was improved by CUR treatment compared with GM group judged by the decrease of the kidney weights to 1.58 ± 0.12, 1.51 ± 0.03 and 1.57 ± 0.05 g and kidney weight per body weight ratios to 4.75 ± 0.39, 4.39 ± 0.14 and 4.56 ± 0.14 mg/g at doses of 100, 200 and 300 mg/kg, respectively. Furthermore, CUR pre-treatment also improved renal function. Several nephrotoxicity hallmarks were restored including serum BUN lowered to 28.17 ± 0.87, 23.50 ± 0.96 and 22.83 ± 0.60 mg/dL and creatinine levels decreased to 0.69 ± 0.01, 0.50 ± 0.03 and 0.50 ± 0.03 mg/dL while urine creatinine increased to 40.09 ± 2.04, 54.51 ± 2.44 and 54.91 ± 2.45 mg/dL and Ccr augmented to 0.97 ± 0.07, 1.71 ± 0.04 and 1.82 ± 0.19 mL/min at doses of 100, 200 and 300 mg/kg, respectively. These results clearly indicated that CUR pre-treatment could potentially protect renal dysfunction induced by GM.

**Table 1.** Effect of curcumin on body weight and kidney weight changes and biomarkers of kidney function.

Parameters	CON	GM	GM + CUR 100	GM + CUR 200	GM + CUR 300	CUR
BW (g)	358 ± 2	273 ± 9*	333 ± 6^†^	346 ± 8^†^	343 ± 4^†^	354 ± 4^†^
KW (g)	1.40 ± 0.04	2.00 ± 0.06*	1.58 ± 0.12^†^	1.51 ± 0.03^†^	1.57 ± 0.05^†^	1.34 ± 0.03^†^
KW/BW (mg/g)	3.92 ± 0.11	7.41 ± 0.44*	4.75 ± 0.39^†^	4.39 ± 0.14^†^	4.56 ± 0.14^†^	3.67 ± 0.05^†^
Urine output (mL/h)	0.85 ± 0.05	1.61 ± 0.06*	1.03 ± 0.10^†^	0.94 ± 0.06^†^	0.99 ± 0.09^†^	0.92 ± 0.05^†^
BUN (mg/dL)	21.17 ± 0.83	35.00 ± 0.73*	28.17 ± 0.87*†	23.50 ± 0.96^†,‡^	22.83 ± 0.60^†,‡^	21.33 ± 1.14^†,‡^
SCr (mg/dL)	0.46 ± 0.02	0.95 ± 0.04*	0.69 ± 0.01*^,†^	0.50 ± 0.03^†,‡^	0.50 ± 0.03^†,‡^	0.42 ± 0.01^†,‡^
UCr (mg/dL)	60.26 ± 5.23	29.01 ± 2.23*	40.09 ± 2.04*	54.51 ± 2.44^†,‡^	54.91 ± 2.45^†,‡^	56.38 ± 2.70^†,‡^
CrCl (mL/min)	1.82 ± 0.10	0.83 ± 0.09*	0.97 ± 0.07*	1.71 ± 0.04^†,‡^	1.82 ± 0.19^†,‡^	2.04 ± 0.11^†,‡^

BW: body weight; KW: kidney weight; KW/BW: kidney weight to body weight; BUN: blood urea nitrogen; SCr: serum creatinine; UCr: urine creatinine; CrCl: creatinine clearance; CON: control; GM: gentamicin; GM + CUR 100: gentamicin plus curcumin at doses of 100 mg/kg; GM + CUR 200: gentamicin plus curcumin at doses of 200 mg/kg; GM + CUR 300: gentamicin plus curcumin at doses of 300 mg/kg; CUR: curcumin alone at doses of 200 mg/kg.

Data are mean ± SEM of six rats.

* *p* < 0.05 vs. CON.

† *p* < 0.05 vs. GM.

‡ *p* < 0.05 vs. GM + CUR 100.

### Effect of curcumin pre-treatment on oxidative stress status

**Figure 2.**
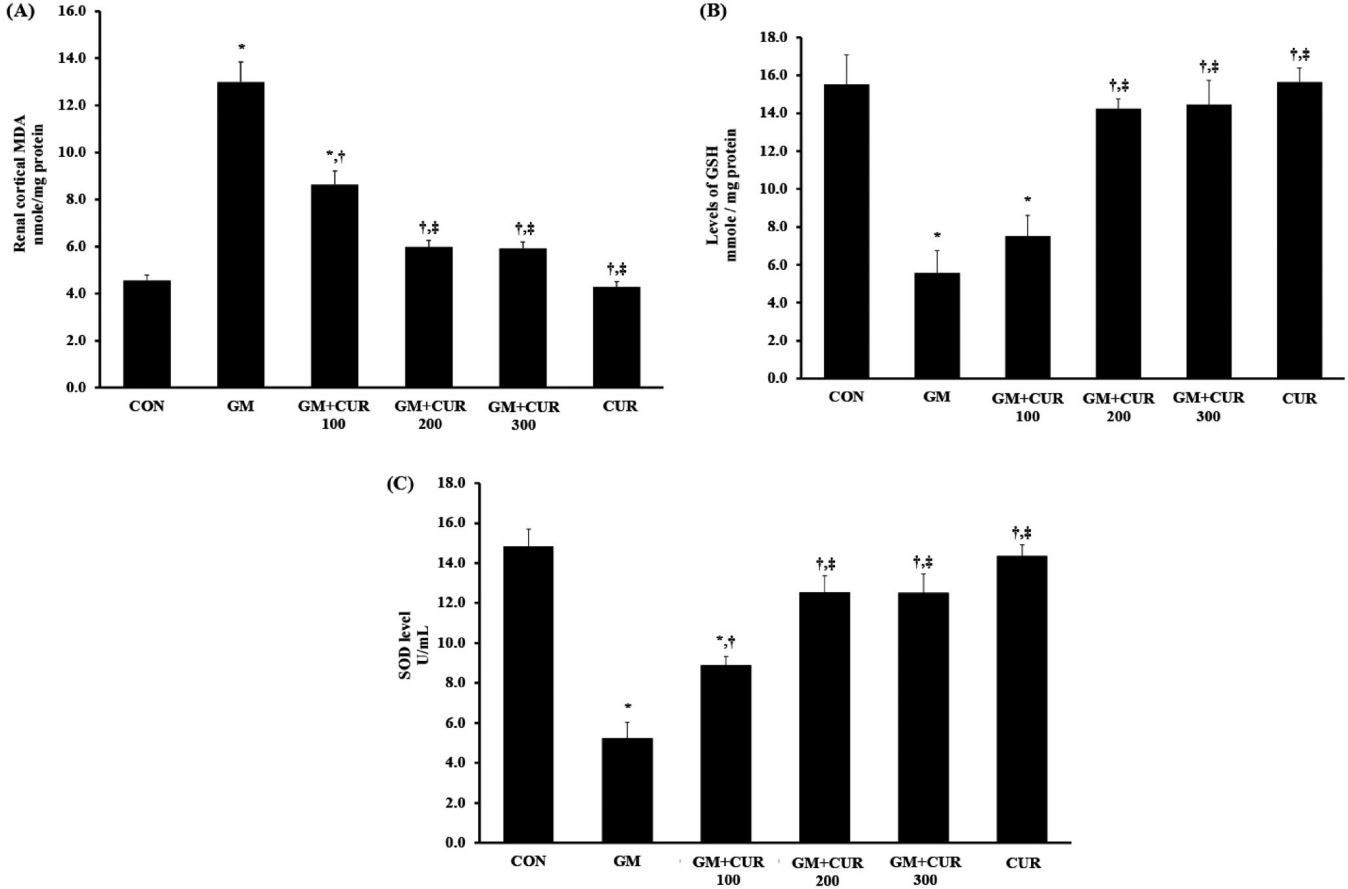
Effect of curcumin on renal cortical MDA (A), GSH (B) and SOD (C) levels. The levels MDA, GSH and SOD were determined after treatment of curcumin (CUR) at doses of 100, 200 and 300 mg/kg in gentamicin-treated group (GM) compared to control (CON). Data are mean ± SEM of six rats. *p < 0.05 vs. CON; ^†^*p* < 0.05 vs. GM. ^‡^*p* < 0.05 vs. GM + CUR 100.

In concordance with the previous report (Jaikumkao et al. 2016), our study demonstrated that the GM treatment dramatically induced the renal oxidative stress. The rat with GM treatment had a significant increase of renal cortical MDA (13.00 ± 0.85 nmol/mg protein) compared to the CON group (4.55 ± 0.25 nmol/mg protein) (Figure 2(A)). In accompanied with the MDA alteration, the significant reduction of renal cortical GSH (from 15.54 ± 1.57 to 5.59 ± 1.16 mmol/mg protein) and SOD (from 14.81 ± 0.88 to 5.22 ± 0.81 U/mL) was observed (Figure 2(B,C)) which emphasized the effects of GM on renal oxidative stress. Interestingly, CUR pre-treatment apparently alleviated renal cortical oxidative stress in GM-treated rats. Pre-treatment of CUR showed the dose-dependent effects to decrease the MDA to 8.63 ± 0.59 and 5.98 ± 0.29 nmol/mg protein at the doses of 100 and 200 mg/kg, respectively (Figure 2(A)) whereas these treatments could potentially increase the GSH to 7.51 ± 1.11 and 14.24 ± 0.51 mmol/mg protein as well as the SOD to 8.88 ± 0.49 and 12.53 ± 0.83 U/mL, respectively (Figure 2(B,C)). Notably, treatment with high dose 300 mg/kg provided the comparable activity to those with 200 mg/kg treatment. Thus, CUR at the dose of 200 mg/kg was selected for the subsequent experiments.

### Effect of curcumin pre-treatment on kidney injury and renal fibrosis

**Figure 3.**
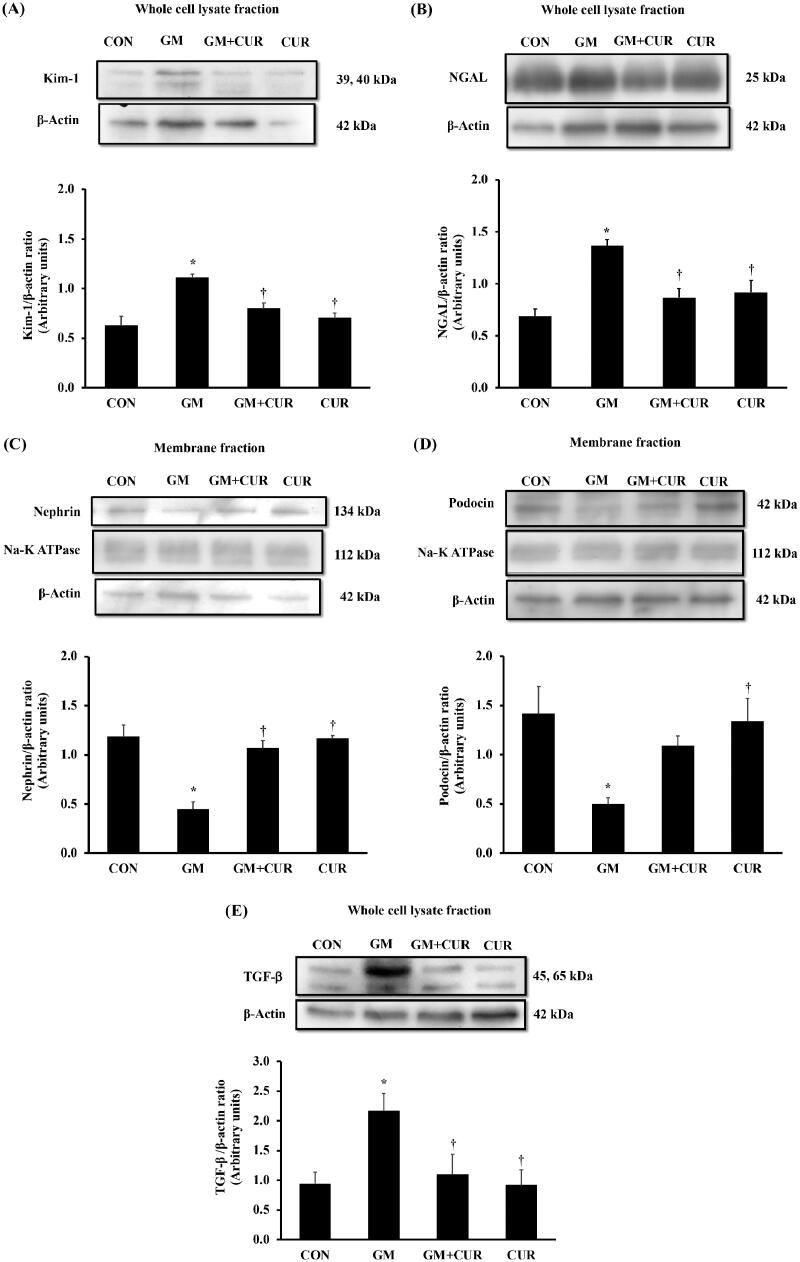
Effect of curcumin on Kim-1, NGAL, nephrin, podocin and TGF-β expression in the renal cortex. The expression of Kim-1 (A), NGAL (B), nephrin (C) and podocin (D) TGF-β (3E) was determined after treatment of curcumin (CUR) at doses of 200 mg/kg in gentamicin-treated group (GM) compared to control (CON) using Western blot analysis. Bar graphs indicate means ± SEM (*n* = 5). *p < 0.05 vs. CON; ^†^*p* < 0.05 vs. GM.

It has been well-known that ROS production and oxidative stress promote kidney injury and provoke the process of fibrosis, causing subsequent loss of kidney function (Hosohata 2016). Herein, we demonstrated the consequence of GM treatment on the renal injury and its involvement in renal fibrosis promotion by measuring the KIM-1, NGAL and TGF-β. The expression levels of KIM-1 and NGAL as markers of tubular injury and nephrin and podocin as markers of glomerular injury in the renal cortex are shown in Figure 3(A,B). The result showed that the GM-treated rats had increased KIM-1 and NGAL expression with the normalized expression of 1.11 ± 0.04 and 1.37 ± 0.06 compared to those CON group with normalized expression of 0.63 ± 0.09 and 0.69 ± 0.07, respectively. Moreover, it caused the significant reduction of nephrin expression from 1.19 ± 0.12 (CON) to 0.45 ± 0.08 in addition to podocin from 1.42 ± 0.28 (CON) to 0.49 ± 0.07 (Figure 3(C,D)). Pre-treatment of CUR potentially rescued the cells from the injury by decreasing the expression of KIM-1 (0.80 ± 0.06) and NGAL (0.87 ± 0.09) and increasing expressions of nephrin (1.07 ± 0.08) and podocin (1.09 ± 0.10) (Figure 3(A–D)). Additionally, we investigated the level of TGF-β which plays a pivotal role in the promotion of renal fibrosis. Concordantly, treatment of GM dramatically increased the expression of TGF-β with the normalized expression of 2.17 ± 0.29 compared to CON group (0.95 ± 0.19) but it could be significantly rescued by CUR pre-treatment to 1.10 ± 0.34 (Figure 3(E)).

### Effect of curcumin pre-treatment on ER stress pathway

**Figure 4.**
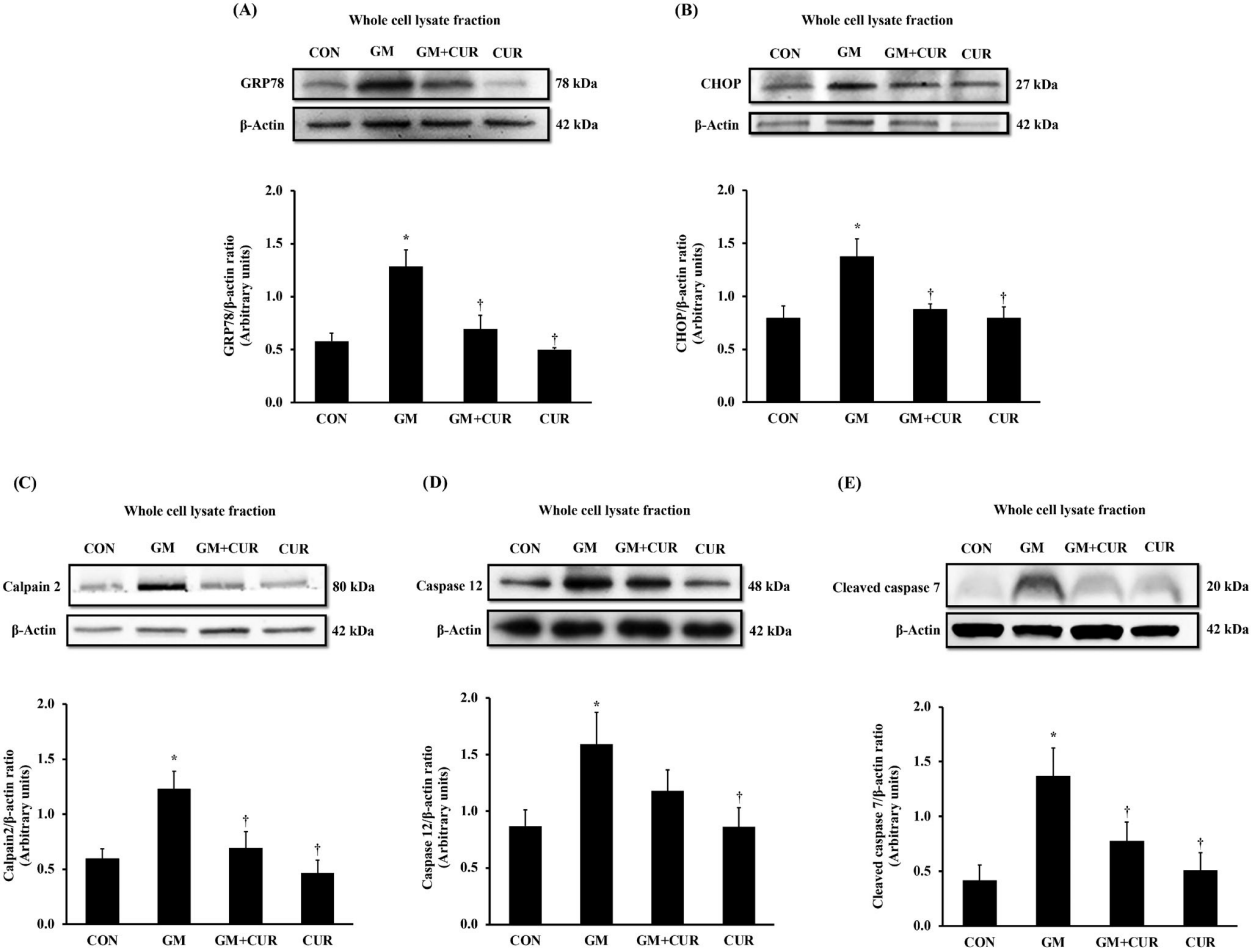
Effect of curcumin on GRP78, CHOP, calpain 2, caspase 12 and cleaved caspase-7 expression in the renal cortex. The expression of GRP78 (A), CHOP (B), calpain-2 (C), caspase-12 (D) and cleaved caspase-7 (E) proteins was determined after treatment of curcumin (CUR) at doses of 200 mg/kg in gentamicin-treated group (GM) compared to control (CON) using Western blot. Bar graphs indicate means ± SEM (*n* = 5). *p < 0.05 vs. CON; ^†^*p* < 0.05 vs. GM.

ER is a subcellular target of toxic compounds involved in drug- or chemical-induced nephrotoxicity (Cribb et al. 2005), and the enhanced production of ROS and subsequent oxidative stress are integral components of ER stress. We investigated the alteration of proteins involving in the ER stress after the GM treatment. As shown in Figure 4(A–E), the renal cortical protein expressions of ER stress markers were significantly elevated compared with rats in the CON group including GRP78 (from 0.58 ± 0.08 to 1.29 ± 0.16), CHOP (from 0.79 ± 0.11 to 1.37 ± 0.17), calpain-2 (from 0.60 ± 0.09 to 1.23 ± 0.16), caspase-12 (from 0.87 ± 0.14 to 1.59 ± 0.28) and cleaved caspase-7 (from 0.41 ± 0.14 to 1.37 ± 0.26). In accompanied with the previous experiment, these changes of ER stress markers were reversed by CUR pre-treatments (GRP78: 0.70 ± 0.13, CHOP: 0.88 ± 0.05, calpain-2: 0.69 ± 0.15, caspase-12: 1.18 ± 0.19, cleaved caspase-7: 0.78 ± 0.17). These results suggested that CUR pre-treatment inhibited the GM-induced ER stress activation.

### Effect of curcumin pre-treatment on apoptotic pathway

**Figure 5.**
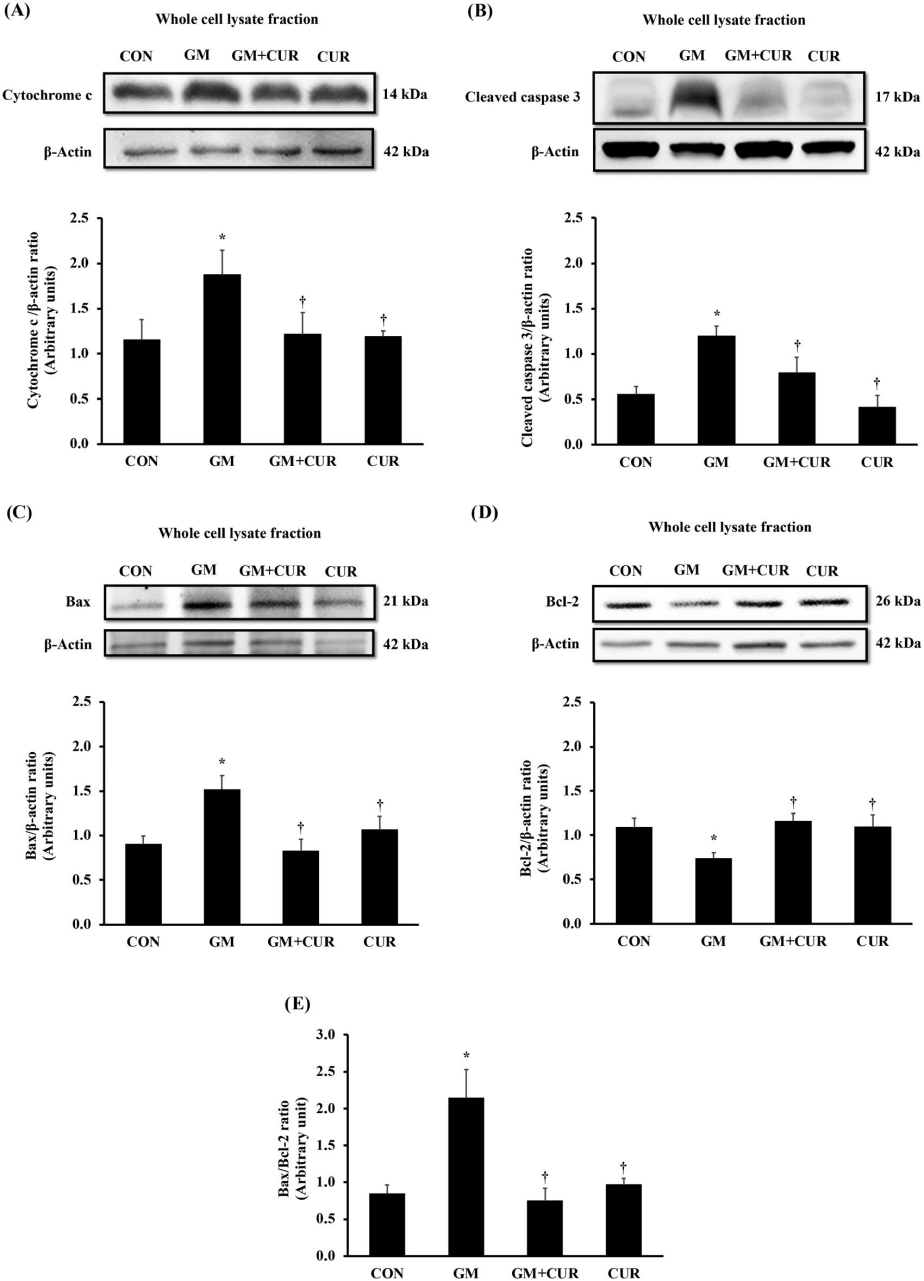
Effect of curcumin on cytochrome c, cleaved caspase 3, Bax and Bcl-2 expression in the renal cortex. The expression of cytochrome-c (A), cleaved caspase-3 (B), Bax (C), Bcl-2 (D) proteins and densitometric analysis of the ratio of Bax to Bcl-2 (E) was determined after treatment of curcumin (CUR) at doses of 200 mg/kg in gentamicin-treated group (GM) compared to control (CON) using Western blot analysis. Bar graphs indicate means ± SEM (*n* = 5). *p < 0.05 vs. CON; ^†^*p* < 0.05 vs. GM.

To investigate the GM effect on promoting the renal cell death, the alteration of proteins involving in apoptosis pathway was monitored. The normalized expression of renal cortical cytochrome-c (1.88 ± 0.27) and cleaved caspase-3 proteins (1.20 ± 0.11) was significantly increased in the GM-treated rats, compared with rats in control in groups (1.15 ± 0.22 and 0.55 ± 0.09, respectively) (Figure 5(A,B)). These were accompanied by a marked decrease in renal cortical anti-apoptotic Bcl-2 protein expression (from 1.09 ± 0.10 to 0.74 ± 0.06), together with significant increase in pro-apoptotic Bax protein (from 0.90 ± 0.10 to 1.52 ± 0.16), and the Bax/Bcl-2 ratio (from 0.85 ± 0.11 to 2.15 ± 0.38) (Figure 5(C–E)). Curcumin pre-treatment apparently counteracted the cell death by lowering cytochrome-c (1.22 ± 0.24) and cleaved caspase-3 (0.79 ± 0.17). Moreover, the treatment restored the expression of anti-apoptotic Bcl-2 (1.16 ± 0.09) whereas lowered the apoptotic Bax (0.83 ± 0.13) and Bax/Bcl-2 ratio (0.76 ± 0.16) to normal levels. These results demonstrate the anti-apoptotic potential of CUR to rescue the GM-induced cell death.

## Discussion

Nephrotoxicity defined as a rapid decline in kidney function by numerous factors such as chemicals industrial, environmental toxic agent and especially drugs. Gentamicin is an aminoglycoside antibiotic that may lead to nephrotoxicity by induction of oxidative stress (Al-kuraishy et al. 2019). The significant increases in serum BUN and creatinine levels, along with the decreased urine creatinine and Ccr, reflected renal dysfunction in GM-treated rats in this study. Furthermore, elevated expressions of KIM-1 and NGAL, along with declines in nephrin and podocin that correlated with enhanced TGF-β expression, were observed in GM administration, indicating renal injury and fibrosis. Previous studies have reported that GM treatment showed the changes in the expression of KIM-1 and NGAL before increases in serum creatinine and urea levels (Ichimura et al. 2004; Luo et al. 2016). Although the functions of these proteins are unclear, they are closely related to apoptosis (Prozialeck et al. 2009) and involved in the repair, proliferation and regeneration responses to toxicity (Huo et al. 2010; Soni et al. 2010). Meanwhile, the nephrin and podocin are necessary for the proper function of the glomerular filtration barrier (Welsh and Saleem 2010), and associated with the development of proteinuria (Nakatsue et al. 2005). It is well established that ROS and the subsequent oxidative stress play an important role in the development of nephrotoxicity and are involved in the mechanisms that decrease of glomerular filtration rates and cause glomerular and tubular injury, along with renal dysfunction (Lopez-Novoa et al. 2011). Study of GM-induced nephrotoxic rats demonstrated enhanced production of hydrogen peroxides, superoxide anions and hydroxyl radicals in the renal cortical mitochondria (Nagai and Takano 2004; Nagai 2006), concomitant with the reduced efficiency of antioxidant enzymes such as SOD, catalase, glutathione peroxidase and GSH, indicating the involvement of oxidative stress in nephrotoxicity (Du and Yang 1994; Martinez-Salgado et al. 2002; Balakumar et al. 2010). In this study, the significant increase in renal cortical MDA levels, with apparent decreases in GSH and SOD in GM-treated rats, were consistent with the previous report demonstrating elevated serum creatinine and BUN levels associated with the overproduction of free radicals in GM-induced nephrotoxicity (Balakumar et al. 2010). Interestingly, CUR treatment effectively decreased renal cortical MDA levels and significantly increased GSH and SOD in GM-treated rats. These were accompanied by significant decreases in BUN and creatinine levels, as well as restoring the expression of KIM-1, NGAL, nephrin, podocin and TGF-β proteins. It is well established that antioxidant activity of CUR is mainly from the phenolic OH group, although a small fraction may be due to the CH_2_ site (Priyadarsini et al. 2003). Curcumin eliminates the hydroxyl radical, superoxide radical, singlet oxygen, nitrogen dioxide and NO (Ruby et al. 1995; Sreejayan and Rao 1997) and has been shown to inhibit hydrogen peroxide induced oxidative injury in a renal cell line (Farombi and Ekor 2006) and prevented reduced activity of antioxidant enzymes including glutathione peroxidase and SOD in rat remnant kidney (Tapia et al. 2012). These results demonstrated that CUR inhibited oxidative stress and restoration of the antioxidant enzymes leading to improved renal function and rescued kidney damage in GM-induced nephrotoxicity.

Recently, oxidative stress has been shown to be an important initiator and major contributor to ER stress (Yuzefovych et al. 2013). Accumulation of GM in ER of the proximal tubular cells causes excessive ROS production, which leads to a redox imbalance and may trigger ER stress and the subsequent ER stress response (ESR) (Szegezdi et al. 2006; Malhotra and Kaufman 2007). In this study, the significant increases in calpain-2, caspase-12, GRP78, CHOP, cytochrome-c, cleave caspase-3 and pro-apoptotic Bax expression, along with the decreased expression of anti-apoptotic Bcl-2 in renal cortical tissues in GM-treated rats were consistent with the previous study demonstrating that accumulation of GM treatment induced ER stress resulting in activation of ER-mediated cell death markers (Jaikumkao et al. 2016). The increased expression of renal cortical CHOP promoted the translocation of Bax from cytosol to mitochondria, leading to a loss of mitochondrial membrane potential and subsequent release of cytochrome-c and activation of caspase-3 that causes apoptosis (Kitanaka et al. 1997; Pastorino et al. 1998; Nakamura et al. 2000). Also, increased cytosolic calcium levels during ER stress promoted mobilization of calpain to the ER, resulting in activation of caspase-12 that subsequently triggered the activation of caspase-7 (Nakagawa and Yuan 2000; Hitomi et al. 2004). Interestingly, the renal cortical ER stress markers accompanied by renal apoptosis were ameliorated in GM-treated rats with CUR pre-treatment. These results were in line with the previous report demonstrating that CUR ameliorated high glucose-induced neural tube defects by suppressing ER stress, caspase activation and apoptosis (Afrin et al. 2015). Moreover, CUR treatment also prevented cell death induced by H_2_O_2_ (Wang et al. 2016). It might be suggested that CUR attenuated ER stress-induced apoptosis in GM-induced nephrotoxicity, probably associated with the antioxidative activity.

## Study limitation

The finding in this present study revealed the therapeutic effect of CUR to rescue nephrotoxicity in animal model. However, the further investigation, especially a clinical trial, is necessary to provide the data regarding the safety and therapeutic efficiency in human.

## Conclusions

The GM-induced nephrotoxicity was contributed by the increasing of oxidative stress, ER stress activation which eventually resulted in the apoptosis cell death. These events were restored by the pre-treatment of CUR based on its activities to reverse several hallmarks of nephrotoxicity including serum creatinine, BUN, Ccr, MDA, GSH and SOD in addition to restoration of the expression levels of ER stress markers (GRP78, CHOP, calpain-2, caspase-12 and cleaved caspase-7) and apoptotic protein biomarkers (cytochrome-c, cleaved caspase-3, BAX and Bcl-2). These findings emphasized the potential therapeutic use of CUR to be developed as an alternative treatment for nephrotoxicity.

## Data Availability

The data that support the findings of this study are available from the corresponding author upon reasonable request.
